# Mapping of neuroinflammation-induced hypoxia in the spinal cord using optoacoustic imaging

**DOI:** 10.1186/s40478-022-01337-4

**Published:** 2022-04-11

**Authors:** Marta Ramos-Vega, Pontus Kjellman, Mihail Ivilinov Todorov, Tekla Maria Kylkilahti, B. Thomas Bäckström, Ali Ertürk, Chris Denis Madsen, Iben Lundgaard

**Affiliations:** 1 Department of Experimental Medical Science, Lund University, 223 62 Lund, Sweden; 2 Wallenberg Center for Molecular Medicine, Lund University, 223 62 Lund, Sweden; 3 Division of Translational Cancer Research, Department of Laboratory Medicine, Lund University, 22381 Lund, Sweden; 4 Institute for Tissue Engineering and Regenerative Medicine (ITERM), Helmholtz Zentrum München, 85764 Neuherberg, Germany; 5 Institute for Stroke and Dementia Research (ISD), University Hospital, Ludwig-Maximilians-Universität LMU, 81377 Munich, Germany; 6 Munich Cluster for Systems Neurology (SyNergy), Munich, 81377 Germany; 7 Department of Clinical Sciences, Department of Neurosurgery, Lund University, 221 84 Lund, Sweden

**Keywords:** Neuroinflammation, Hypoxia, Optoacoustic imaging, Light sheet fluorescence microscopy, EAE, Multiple sclerosis

## Abstract

Recent studies suggest that metabolic changes and oxygen deficiency in the central nervous system play an important role in the pathophysiology of multiple sclerosis (MS). In our present study, we investigated the changes in oxygenation and analyzed the vascular perfusion of the spinal cord in a rodent model of MS. We performed multispectral optoacoustic tomography of the lumbar spinal cord before and after an oxygen enhancement challenge in mice with experimental autoimmune encephalomyelitis (EAE), a model for MS. In addition, mice were transcardially perfused with lectin to label the vasculature and their spinal columns were optically cleared, followed by light sheet fluorescence microscopy. To analyze the angioarchitecture of the intact spine, we used VesSAP, a novel deep learning-based framework. In EAE mice, the spinal cord had lower oxygen saturation and hemoglobin concentration compared to healthy mice, indicating compromised perfusion of the spinal cord. Oxygen administration reversed hypoxia in the spinal cord of EAE mice, although the ventral region remained hypoxic. Additionally, despite the increased vascular density, we report a reduction in length and complexity of the perfused vascular network in EAE. Taken together, these findings highlight a new aspect of neuroinflammatory pathology, revealing a significant degree of hypoxia in EAE in vivo that is accompanied by changes in spinal vascular perfusion. The study also introduces optoacoustic imaging as a tractable technique with the potential to further decipher the role of hypoxia in EAE and to monitor it in MS patients.

## Introduction

Multiple sclerosis (MS) is a disabling neurodegenerative disease of the central nervous system (CNS) that is thought to be caused by an autoimmune attack to myelin proteins by the immune system, leading to demyelination of nerve fibers and axonal damage [9]. Most of the experimental research on MS has taken advantage of the animal model EAE (experimental autoimmune encephalomyelitis) to study the importance of immune and inflammatory components, however, the disease is still not well understood [10, 17]. Over the past few years, hypoxia or low tissue oxygenation has emerged as a new potential factor contributing to neuroinflammation in the disease [23].

With the goal to uncover new disease mechanisms in MS, recent studies have demonstrated disturbances related to tissue oxygenation within lesions in MS patients [1, 34, 43] and in EAE models [11, 22, 32]. Since hypoxia can elicit inflammation by recruiting cells to the affected area, some researchers have suggested the possible existence of a “hypoxia–inflammation” cycle [44]. Furthermore, MS lesions tend to occur in watershed regions in the CNS with poor perfusion [12], which supports the hypothesis that poor distribution of oxygen may contribute to the pathology. Thus, hypoxia is emerging both as a biomarker of pro-inflammatory activity and as a potential therapeutic target in MS.

In other fields, the study of oxygenation has advanced with the advent of non-invasive optoacoustic imaging (OA) to study in vivo oxygen dynamics in animal models [3, 19, 28, 41]. Techniques such as multispectral optoacoustic tomography (MSOT) provide information about the distribution of the tissue optical absorption in real time at multiple wavelengths. Taking advantage of the distinct absorption spectra of oxy- and deoxy-hemoglobin, it is possible to obtain dynamic images and measurements of hemoglobin concentrations and oxygen saturation levels in tissue [27].

In this study, we present for the first time a robust and reproducible readout of the spinal cord oxygenation and hemodynamics by means of optoacoustic imaging using MSOT. We confirm the presence of hypoxia in the lumbar spinal cord of EAE and show that the administration of high concentration of oxygen relieves this hypoxia. Since the concept of tissue oxygenation is tightly linked to the vasculature, we also inspected changes in the vasculature of the spinal cord by means of vascular staining and light sheet fluorescence microscopy (LSFM). By applying a newly developed deep learning-based framework, VesSAP (Vessel Segmentation & Analysis Pipeline) [40], we reveal a reduction in the perfused vascular network in the spinal cord of EAE.

This study adds to a fast-growing field of research investigating the role of hypoxia and declined vascular function in EAE, and also introduces OA as a suitable noninvasive and simple method to study the effects of prospective therapeutical approaches targeting tissue hypoxia in EAE and MS.

## Materials and methods

### Animals

Adult female SJL/J mice purchased from Janvier Labs (Le Genest-Saint-Isle, France) were housed according to the regulations of Lund University and compliant with the international ARRIVE guidelines on experimental animal research. All experiments were conducted in accordance with the Swedish National Institute of Health for the Care and Use for Laboratory animal (License number 10258/2018, approved by the local Ethical Committee for Animal Research).

### EAE induction

To induce EAE, POWER-Kits™ were purchased from BTB Emulsions (Malmö, Sweden) and Complete Freund’s Adjuvant (CFA)/antigen emulsions were prepared according to the manufacturer’s recommendations (see https://btbemulsions.com/). Mice were injected subcutaneously at the flank on the back of the mouse with 200 µl (100 µl on each side) consisting of 75 μg mouse PLP_139-151_ peptide (synthesized by Innovagen, Lund, Sweden) emulsified in CFA (Sigma-Aldrich, F5881) with an additional 200 μg Mycobacterium tuberculosis H37RA (Difco, Detroit, MI). Bordetella pertussis toxin (Sigma-Aldrich, P2980) was injected 80 ng/200 μl in pertussis toxin buffer (Ca^2+^/Mg^2+^ free PBS with the addition of 0.5 M NaCl and 0.01% Triton X-100) intraperitoneally (100 µl on each side) on day 0 (2 h after immunization) and again after 24 h. The mice were observed daily for clinical signs and scores were assigned based on the following scale: 0.5, partial tail weakness; 1, limp tail; 1.5, limp tail and hind leg inhibition; 2, limp tail and weakness of hind legs; 2.5, limp tail and dragging of one hind leg or both legs slightly dragging at the feet; 3, paralysis of the hindlegs; 3.5, all the previous and unable to right itself or hind quarters are flat like a “pancake”; 4, all the previous and partial front leg paralysis.

The average onset of disease was on day 9–10 post immunisation (DPI) and mice were used for live imaging and/or perfusion at the peak of disease, DPI 11–13.

### Multispectral optoacoustic tomography (MSOT)

Multispectral optoacoustic tomography was performed using an inVision 512-echo (iThera Scientific GmbH, Munich, Germany) small animal imaging system [30]. Briefly, a tunable optical parametric oscillator (OPO) pumped by an Nd:YAG laser provides excitation pulses with a duration of 9 ns at wavelengths from 680 to 980 nm at a repetition rate of 10 Hz with a wavelength tuning speed of 10 ms and a peak pulse energy of 100 mJ at 730 nm. Ten arms of a fiber bundle provide even illumination of a ring-shaped light strip of approx. 8 mm width. For ultrasound detection, 512 toroidally-focused ultrasound transducers with a center frequency of 5 MHz (60% bandwidth), organized in a concave array of 270-degree angular coverage and a radius of curvature of 4 cm, were used. In addition to optoacoustic imaging, the system is also capable of interleaved naturally co-registered reflectance ultrasound computed tomography (RUCT) image acquisition.

Mice (EAE n = 11, control n = 13) were anaesthetized with isoflurane (4% for induction, 2–2.3% for maintenance) and placed on a heating pad. The hair of the midsection was removed by electrical clippers and epilation cream and the animal was subsequently placed in the supine position in the animal holder. Ultrasound gel was applied to the skin to couple it to the polyethylene foil surrounding the mouse and creating a watertight pocket. The animal holder was lowered into the 36 °C water bath of the imaging chamber and the mouse was allowed to acclimatize for 15 min. An axial imaging section of the lumbar part of the spine, approximately 50 mm caudal of the nose, was located and the breathing rate of the mouse was established at 70–80 breaths per minute by adjusting the anesthesia. The breathing rate was determined by counting the breaths during one minute of live imaging. During induction, preparation, and acclimatization the mice were breathing medical air (21% O_2_). Continuous imaging was commenced using 10 wavelengths (700, 730, 750, 760, 770, 800, 820, 840, 850, 880 nm) with an average of 10 pulses per wavelength. In oxygen enhancement (OE) challenge experiments, after imaging for 5 min the breathing air was switched from conventional medical air to 100% O_2_ and after 20 min it was switched back to medical air (21% O_2_) and imaged for 5 more minutes. Thus, animals were continuously imaged for 30 min (5 min at 21% O_2_; 20 min at 100% O_2_; and 5 min at 21% O_2_).

### MSOT data analysis

Acquired signals were spatially and quantitatively resolved with back-projection reconstruction and optoacoustic data was then unmixed using linear regression in viewMSOT software version 4.0 (iThera Scientific) into the relevant individual chromophores (hemoglobin and oxygenated hemoglobin) and its derived components (total hemoglobin (HbT) and MSOT-derived tissue oxygen saturation (SO_2_^MSOT^)). MSOT layers were overlaid on the co-registered anatomical RUCT image to display their spatial distribution in the mouse cross-sectional view. From the ultrasound images a region of interest (ROI) was drawn around the spinal cord, denoted as Total ROI. This Total ROI was further segmented into a Dorsal and Ventral ROI. Due to the low oxygen saturation of the EAE mice’s spinal cord the threshold of total hemoglobin (HbT) was set to 0 for all mice, to maximize the detected signal. The MSOT-derived tissue oxygen saturation (SO_2_^MSOT^) data was exported and compiled in Microsoft Excel.

### Transcardial vascular staining and tissue clearing

Mice (EAE n = 13, control n = 9) were anesthetized with a ketamine-xylazine mix (Ketamine, 100 mg/kg; Xylazine, 20 mg/kg). Shortly before perfusion-fixation, 50 µl of 1 mg/ml Lycopersicon Esculentum lectin conjugated to DyLight649 (DL-1178, VectorLabs) was transcardially injected [5]. After 5 min of circulation animals were transcardially perfusion-fixed with phosphate buffered saline (PBS) containing 5 IU/ml heparin followed by 4% Paraformaldehyde (PFA, in 0.2 M phosphate buffer, pH 7.4) for fixation of the tissue. Spinal columns were harvested and post-fixed overnight in 4% PFA. The iDISCO+ (immunolabeling-enabled three-dimensional imaging of solvent-cleared organs) protocol was carried out as described by Renier et al. (2016). Briefly, the spinal columns were first decalcified in EDTA (10% w/v, pH 8–9, for 24 h). Next, the tissue was dehydrated in increasing methanol/H_2_O series (20%, 40%, 60%, 80%, 100%, 100%, 1 h each), delipidated with methanol/dichloromethane (33%/66% for 3 h) and pure dichloromethane (2 × 15 min), and optically cleared with dibenzyl ether (DBE, 100%) for at least 14 days. Lastly DBE was replaced with ethyl cinnamate (ECi, 100%) at least 7 days prior to imaging.

### Light sheet fluorescence microscopy

Optically cleared spinal columns were imaged using a LaVision UltraMicroscope Blaze light sheet microscope (Miltenyi Biotec) using a 4 × objective (LaVision BioTec MI Plan 4 × /0.35 NA, zoom 0.6×) equipped with an organic solvent compatible immersion corrected dipping cap. The excitation wavelength was 640 nm and the emission filter used was 680/30 nm. The samples were imaged while immersed in ECi in the sagittal plane, from one lateral side to the other, in only the lumbar region of the spinal cord at a z-step size of 3 µm with ImspectorPro64 (LaVision BioTec, Bielefeld, Germany) software using both left and right-side illumination and single field of view. To avoid defocus, we used a 6-step sequential shifting of the focal position of the light sheet per plane and side. The thinnest point of the light sheet was 5 µm.

### Light sheet data analysis

We used the vessel segmentation and analysis pipeline (VesSAP) [40] to quantify the vasculature changes in the lumbar spinal cord by firstly manually defining a mask for the CNS tissue only separating it from the vertebra and surrounding vasculature. Next, we ran the segmentation, preprocessing and feature extraction to obtain the total vessel length (sum of vessel centerline voxels), bifurcation density (sum of segmentation skeleton bifurcations), and average radius of vessels (distance of all centerline voxels to the nearest segmentation mask). All measures were then corrected by a constant (described as the in vivo space in [40]) to account for shrinkage due to fixation and clearing.

### Immunohistochemistry

We used immunostaining to detect the glucose transporter 1 (Glut1). Optically cleared spinal columns (n = 6 control, n = 5 EAE) were rehydrated in decreasing methanol/H_2_O series (100%, 80%, 60%, 40%, 20%, 1 h each) and finally washed in 1 × PBS overnight, in order to partially reverse the clearing. Then, the lumbar region of the spinal cord was dissected out from the vertebral column and sectioned with a vibratome (100 µm thickness). Selected coronal lumbar spinal cord sections were histochemically stained. Briefly, a blocking solution (0.3% Triton X-100, 5% BSA, and 1% serum) was applied for one hour at room temperature (RT) to minimize unspecific antibody binding. Then, the primary antibody anti-Glut1 (MABS132, 1:250) was allowed to label the tissue overnight at 4 ◦C under gentle shaking. The next day, sections were washed three times with 1 × PBS before incubating with the secondary antibody (Donkey Anti-mouse 488, Invitrogen, 1:500) for 90 min at RT under gentle shaking. Following that, three washes with 1 × PBS, 40,6-diamidin-2-fenilindolo (DAPI, 1:1000) were performed before the sections were mounted.

### Image acquisition and analysis of immunolabeled samples

Mounted spinal cord sections were imaged using a Nikon Ti2 Eclipse microscope at 10 × magnification (Plan Apo 10 × /0.45 NA) for whole section visualization and a Nikon A1RHD confocal microscope at 20 × magnification (Plan Apo 20 × /0.75 NA) and zoom 3 × for detailed imaging of the stained blood vessels. From each animal, three lumbar slices on average were imaged and analyzed. The confocal images were analyzed using the software Fiji (NIH, version 2.0.0-rc-69/1.53c). For the analysis of the number of blood vessels/mm^2^, the vessels were counted manually in each image (3 images per animal on average) on randomly selected sites. For the analysis of the percentage of Glut1^+^ blood vessels labeled by lectin perfusion, Glut1-labeled vessels were identified and the presence of lectin in each vessel was assessed as perfused or not perfused with lectin.

### Experimental design and statistical analysis

All statistical analyses were performed using GraphPad Prism 9 (GraphPad Software). Data distribution were tested for normality by visually assessing the histograms. Since the data was normally distributed, two independent groups were compared using unpaired Student’s *t* test. In the few instances, when two paired groups were compared (e.g., Fig. 2E), a paired Student’s *t* test was used. For comparison of two groups at different times (e.g., MSOT OE data), a two-way ANOVA followed by Tukey’s multiple comparison post-hoc test was used. All values are expressed as mean ± SEM unless stated otherwise. N represents number of animals. *P* < 0.05 was accepted as statistically significant.

## Results

### EAE is associated with low oxygen saturation in the spinal cord

**Fig. 1:**
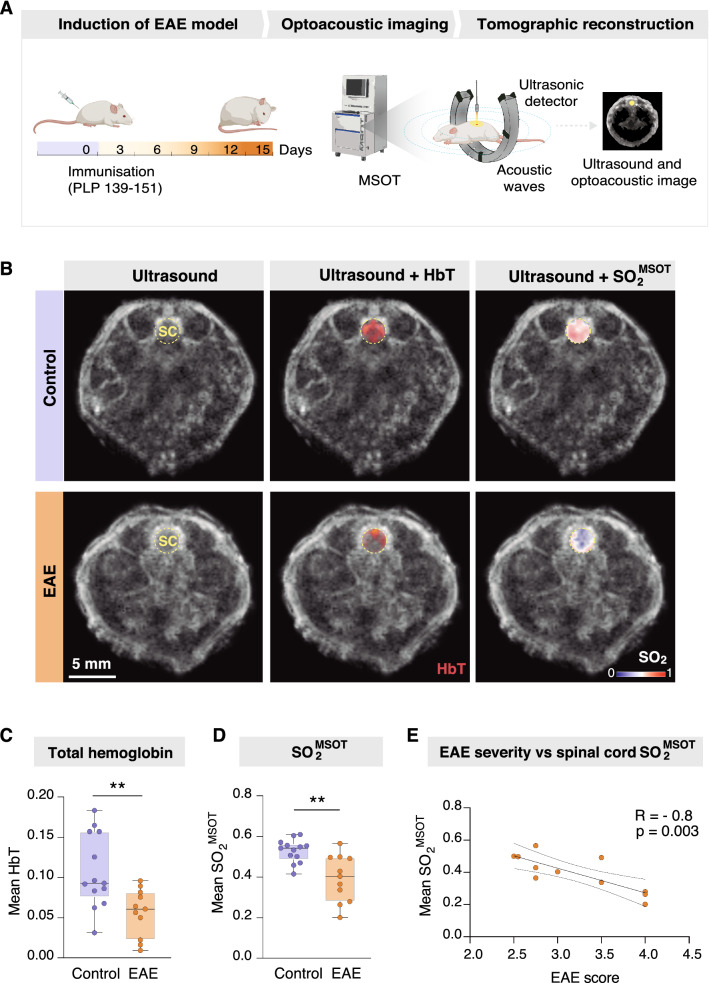
Multispectral optoacoustic tomography reveals low oxygen saturation in the lumbar spinal cord of EAE mice. **A** Schematic representation of the experimental procedure. **B** Representative images of ultrasound, total hemoglobin (HbT) and MSOT-derived oxygen saturation (SO_2_^MSOT^) in the lumbar spinal cord (SC). **C** Total hemoglobin in the spinal cord of EAE and control mice (unpaired *t* test, n = 11–13 per group, median, quartiles, min, max). **D** MSOT-derived oxygen saturation in the spinal cord of EAE and control mice (unpaired *t* test, n = 11–13 per group, (median, quartiles, min, max) **E** Correlation between MSOT-derived oxygen saturation and EAE severity (Pearson’s correlation, r = -0.8, *p* = 0.003, n = 11). *p < 0.05, **p < 0.01. Scale bar in B, 5 mm. Abbreviations: EAE, experimental autoimmune encephalomyelitis; MSOT, multispectral optoacoustic tomography; SC, spinal cord; HbT, total hemoglobin; SO_2_^MSOT^, MSOT-derived oxygen saturation

To induce EAE, adult mice were immunized with the myelin peptide PLP_139-151_ (Fig. 1A). Nine to ten days after immunization, mice started to present the typical symptoms of EAE, e.g., limp tail and motor difficulties. At the acute stage of the disease (DPI 11–13) we investigated the oxygenation levels by means of MSOT imaging (Fig. 1A) in the lumbar spinal cord, which is suspected to be the initial site of inflammation in EAE [2]. Since oxygenated and deoxygenated hemoglobin have different optical absorption wavelengths, using MSOT at multiple wavelengths allowed us to calculate the total haemoglobin concentration (HbT) and the MSOT-derived oxygen saturation (SO_2_^MSOT^) (Fig. 1B). EAE mice exhibited significantly lower HbT in the spinal cord as compared to control mice (Fig. 1C; unpaired *t* test, EAE mean = 0.056, control mean = 0.107, 95% CI of difference =  − 0.08459 to − 0.01773, *p* = 0.004; n = 11–13 per group), indicating poor blood perfusion. In addition, the SO_2_^MSOT^ was significantly decreased in the spinal cord of EAE mice compared to controls (Fig. 1D; unpaired *t* test, EAE mean = 0.529, control mean = 0.394, 95% CI of difference =  − 0.2101 to − 0.05968, *p* = 0.001; n = 11–13 per group). The decrease in SO_2_^MSOT^ strongly correlated with the EAE disease score (Fig. 1E; Pearson r = 0.802, *p* = 0.003, n = 11). These results suggest that the spinal cords of EAE mice are subjected to poor vascular perfusion and hypoxia that correlate with the severity of the disease.

### Oxygen administration improved oxygenation of the spinal cord of EAE

**Fig. 2:**
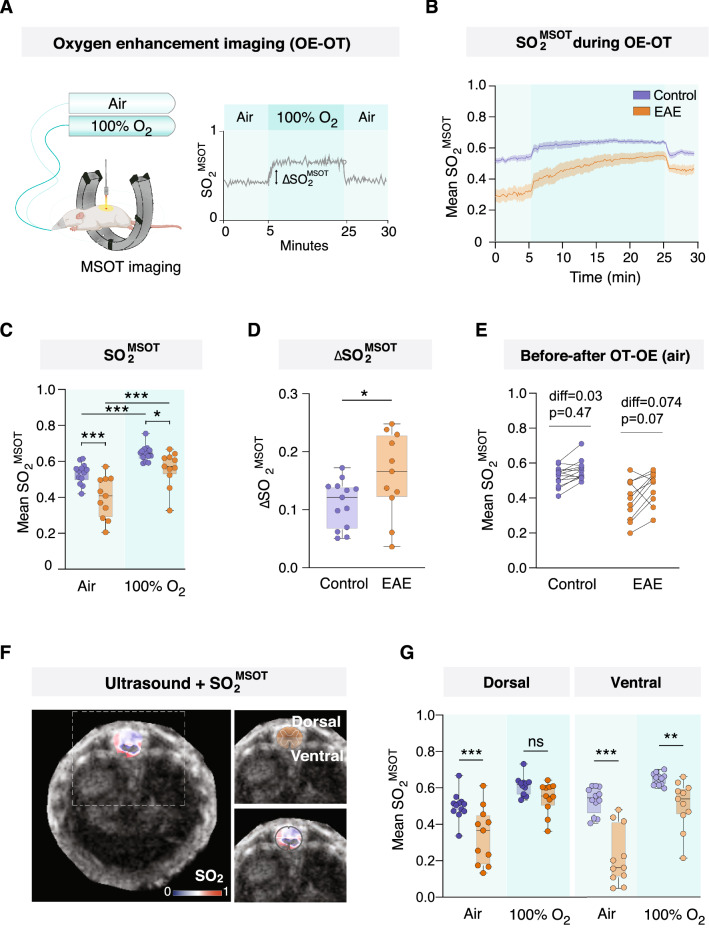
Oxygen administration improved oxygenation of the spinal cord in EAE mice. **A** Diagram depicting the experimental procedure of the oxygen enhancement challenge (OT-OE) using MSOT. **B** Continuous measurement of oxygen saturation in the spinal cord of EAE and control mice during OE-OT (n = 11–13 per group, mean ± SEM). **C** MSOT-derived oxygen saturation (SO_2_^MSOT^) in the spinal cord of EAE and control mice while breathing medical air (21% oxygen, light blue) and after 15 min breathing 100% oxygen (dark blue) (2-way ANOVA and Tukey post-hoc test, n = 11–13 per group, median, quartiles, min, max). **D** Difference in oxygen saturation (ΔSO_2_^MSOT^) of the spinal cord between breathing air and 100% oxygen in EAE and control mice (unpaired *t* test, n = 11–13 per group, median, quartiles, min, max) **E** SO_2_^MSOT^ in the spinal cord of EAE and control mice while breathing medical air before and after OE-OT (before administering 100% oxygen and after mice stopped breathing 100% oxygen) (2-way ANOVA and Tukey post-hoc test, n = 11–13 per group). **F** Representative images and schematic depiction of dorsal and ventral lumbar spinal cord division. **G** MSOT-derived oxygen saturation in the dorsal and ventral spinal cord of EAE and control mice while breathing medical air (light blue) and after 15 min breathing 100% oxygen (dark blue) (2-way ANOVA and Tukey post-hoc test for dorsal and ventral separately, n = 11–13 per group, median, quartiles, min, max). *p < 0.05, **p < 0.01, ***p < 0.001. Abbreviations: OE-OT, oxygen enhancement challenge; ΔSO_2_^MSOT^, difference in oxygen saturation between air and oxygen phases; diff, statistical difference

We next assessed whether the vasculature in the spinal cord of EAE mice responds to oxygen in similar ways to that of healthy mice. To investigate this question, we used an oxygen enhancement challenge during the optoacoustic tomography (OE-OT) (Fig. 2A), which has been successfully used to report vascular function in tumour studies [41]. The administration of oxygen resulted in clear increases in the MSOT-derived oxygen saturation in the lumbar spinal cord of both EAE and control mice (Fig. 2B). It should be noted that EAE mice took longer to achieve their highest SO_2_ point under 100% oxygen, suggesting a less functional vasculature than in healthy animals. Both EAE and control mice exhibited significantly higher SO_2_^MSOT^ after breathing 100% oxygen for 20 min (Fig. 2C; two-way ANOVA, 95% CI of difference control = − 0.1482 to − 0.07275, *p* < 0.001, 95% CI of difference EAE = − 0.2017 to − 0.1197, *p* < 0.001; n = 11–13 per group). At that point, EAE mice had a comparable SO_2_^MSOT^ to that of controls breathing medical air, but they were not able to achieve the SO_2_^MSOT^ levels of controls breathing 100% oxygen (Fig. 2C; two-way ANOVA, EAE mean = 0.555, control mean = 0.639, 95% CI of difference = 0.007514 to 0.1618, *p* = 0.029; n = 11–13 per group). The change in SO_2_ during the OE-OT, indicated by ΔSO_2_^MSOT^, was slightly larger in EAE mice (Fig. 2D; unpaired *t* test, EAE mean = 0.160, control mean = 0.110, 95% CI of difference = 0.002050 to 0.09836, *p* = 0.04). Interestingly, while healthy animals returned to baseline SO_2_^MSOT^ levels once the exposure to 100% oxygen ended (after OE-OT), EAE animals presented, on average, higher SO_2_^MSOT^ after OE-OT as compared to the initial baseline. Although this difference was not statistically significant (Fig. 2E; two-way ANOVA, 95% CI of difference control = − 0.1106 to 0.03884, *p* = 0.4704; 95% CI of difference EAE = − 0.1554 to 0.007056, *p* = 0.078), it suggests that the changes in SO_2_^MSOT^ derived from the administration of oxygen might be sustained with time in EAE. Lastly, we analysed the differences between the dorsal and ventral halves of the lumbar spinal cord since they differ in function and blood supply (Fig. 2F). We found that the ventral half of the spinal cord was the most affected by hypoxia at baseline (Fig. 2G; two-way ANOVA, 95% CI of difference EAE versus control dorsal = 0.06656 to 0.2580, *p* < 0.001; 95% CI of difference ventral = 0.2093 to 0.4174, *p* < 0.001; n = 11–13). In addition, while the SO_2_^MSOT^ levels in the dorsal region increased to control levels after oxygen administration (Fig. 2G; two-way ANOVA, 95% CI of difference EAE versus control dorsal = − 0.03202 to 0.1594, *p* = 0.243), the ventral half of the spinal cord was not able to reach the control levels with oxygen (two-way ANOVA, 95% CI of difference EAE vs control ventral = 0.03630 to 0.2444, *p* = 0.006). These results suggest that oxygen administration relieves hypoxia in the spinal cord of EAE in general, even though, interestingly, the ventral region remained hypoxic in comparison to control.

### Low oxygenation in the spinal cord is associated with reduced perfusion in EAE

**Fig. 3:**
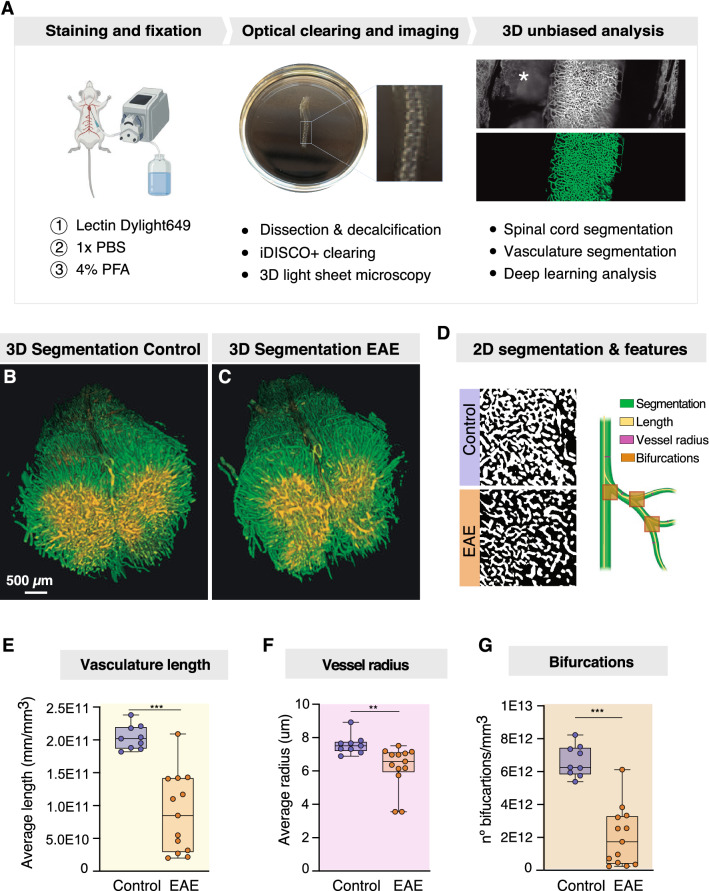
3D imaging shows reduced perfused vasculature network in the spinal cord of EAE mice. **A** Scheme represents the method followed in three steps: 1. Staining with the vascular marker lectin and PFA fixation; 2. Spinal column decalcification and iDISCO+ clearing for 3D imaging of the lumbar cord using LSFM; 3. Segmentation of the spinal cord (excluding the vertebrae, indicated with an asterisk, and surrounding tissue) and deep learning-based segmentation of the blood vessels of the spinal cord followed by 3D reconstruction, feature extraction and analysis. **B** 3D rendering of the lumbar spinal cord segmentation from a control mouse. **C** 3D rendering of the lumbar spinal cord segmentation from a EAE mouse. **D** Left: single slice of vessel segmentation from a representative area from a EAE mouse and a control; right: graphic representation of the features extracted and analyzed from the vessel segmentation. **E**–**G** Quantification of the vasculature length, average vessel radius and number of bifurcations from EAE and control mice (unpaired *t* test, n = 9–13 per group, mean ± SEM). *p < 0.05, **p < 0.01, ***p < 0.001. Scale bar in B and C, 500 μm. Abbreviations: iDISCO, Immunolabeling-enabled three-dimensional imaging of solvent-cleared organs; LSFM, light sheet fluorescence microscopy; VesSAP, Vessel Segmentation & Analysis Pipeline

**Fig. 4:**
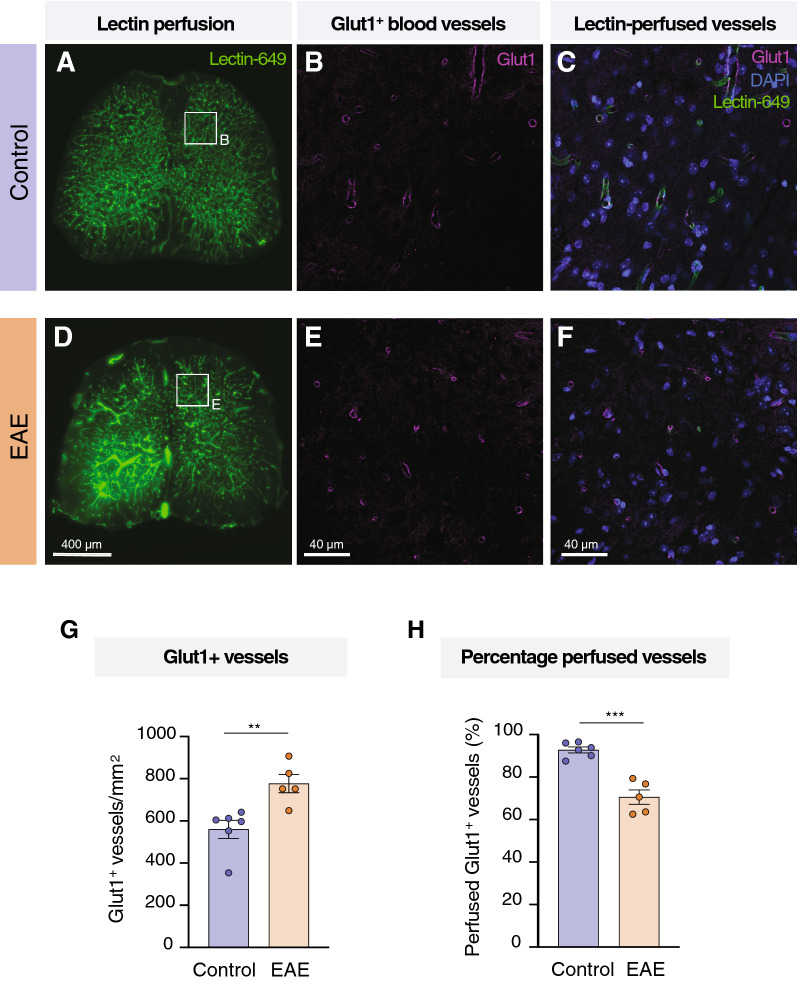
Comparison of lectin-perfused blood vessels and IHC of all blood vessels (Glut1^+^). **A**, **D** Representative fluorescence microscopy images of perfused Lectin-649 signal in lumbar spinal cord sections of control (A) and EAE (D). **B**, **E** Representative images of Glut1^+^ blood vessels stained by IHC and imaged with a confocal microscope in control (B) and EAE (E). **C**, **F** Representative images of lectin-perfused Glut1^+^ vessels in control (C) and EAE (F). **G** Quantification of the number of Glut1^+^ vessels in spinal cord sections from EAE and control mice (unpaired *t* test, n = 5–6 per group, mean ± SEM). **H** Percentage of Glut1^+^ vessels that were lectin-perfused in spinal cord sections of EAE and control mice (unpaired *t* test, n = 5–6 per group, mean ± SEM). *p < 0.05, **p < 0.01, ***p < 0.001. Scale bar in A,D 400 μm and in B-C, E–F 40 μm. Abbreviations: Glut1, Glucose transporter type 1; DAPI, 4′,6-diamidino-2-fenilindol

Lastly, we explored whether spinal hypoxia was associated with changes in the spinal cord vascular network in EAE. To label the perfused vasculature, we injected fluorescently conjugated tomato lectin (Dylight 649) in living mice and allowed its circulation to the vessels, before perfusion fixation (Fig. 3A). We then performed iDISCO+ [35] clearing of the spinal cord and vertebral column and imaged the lumbar spinal cord by means of LSFM (Fig. 3A). Lectin successfully labelled both larger vessels and the extensive microvasculature of the spinal cord (Fig. 3B-C). To enable the extraction of quantitative features of the vascular structure, vessels were segmented in 3D using the deep learning-based framework VesSAP [40]. We then used the segmentation to quantify the vessel length, average vessel radius and the number of bifurcation points (Fig. 3D), which are features that have previously been used to describe the angioarchitecture of the CNS [33]. We found that EAE mice had a marked reduction of the perfused vascular network, indicated by the total vessel length (Fig. 3E; unpaired *t* test, 95% CI of difference = 1.58 × 10^11^ to 7.19 × 10^10^ mm/mm^3^, *p* < 0.001; n = 9–13 per group). In addition, the average vessel radius was significantly smaller in EAE (Fig. 3F; unpaired *t* test, 95% CI of difference = − 2.314 to − 0.3898 μm, *p* = 0.008; n = 9–13 per group). The vessels of EAE mice also exhibited less branching compared to healthy mice (Fig. 3G; unpaired *t* test, 95% CI of difference = 5.97 × 10^12^ ± 3.25 × 10^9^ bifurcations/mm^3^, *p* < 0.001; n = 9–13 per group). Lastly, we sought to confirm that our results in fact reflected reduced perfusion and not a loss of vascular density. To do so, we compared the lectin-perfused vessels with the total number of blood vessels, which were defined by immunostaining of the endothelial cell marker Glut1 on lumbar spinal cord sections. We found that the EAE tissue had a higher Glut1^+^ vessel density than control (Fig. 4G; unpaired *t* test, EAE mean = 777.1, control mean = 560.3; 95% CI of difference = 78.15 to 355.4 vessels/mm^2^, *p* = 0.006; n = 5–6 per group), but the percentage of lectin-perfused Glut1^+^ vessels was nevertheless significantly lower in EAE than in healthy mice (Fig. 4H; unpaired *t* test, EAE mean = 70.59, control mean = 92.81; 95% CI of difference = − 30.01 to − 14.42%, *p* < 0.001; n = 5–6 per group). Taken together, these data argue for an association between low perfusion and oxygenation seen in the spinal cord of EAE and a reduction in the extent and complexity of the perfused vascular network in the spinal cord.

## Discussion

Over the past few years, the relationship between inflammation, vascular dysfunction and hypoxia has become of increasing interest in MS research. So far, reports investigating the therapeutic potential of manipulating oxygen supply in MS patients have been inconclusive [6, 23], similarly to contradictory reports on the CNS vascular integrity of EAE models, which have mostly been based on histology techniques [7, 36, 38]. To shed light on these key questions, we utilized two new imaging approaches, optoacoustic imaging, and light sheet fluorescence microscopy to study the oxygenation and vascular integrity in the spinal cord of EAE mice. This study indicates that the inflamed spinal cord is hypoperfused, resulting in profound effects on oxygen delivery. These findings support recent research indicating that oxygen deficiency exists in the CNS of demyelinating lesions of MS patients and animal models.

From a clinical perspective, studies in MS patients have demonstrated that hemoglobin oxygen saturation in the cerebral cortex is decreased and correlates with clinical disability [43]. Animal studies have also showed the presence of hypoxia in the lumbar spinal cord in rat models of EAE using more traditional methods, such as an oxygen probe and pimonidazole labeling [11, 13, 15]. Our study is the first to use optoacoustic imaging to show the oxygen dynamics of the spinal cord of mice in real time and in a non-invasive way, confirming in vivo that EAE mice have lower oxygen saturation and total hemoglobin concentration in the lumbar spinal cord. It should be noted that Nathoo et al. used susceptibility weighted (SWI) magnetic resonance imaging (MRI) to reveal areas of hypoxia in EAE in the form of focal hypointensities [31, 32]. However, their study also showed that some of these focal hypointensities could instead correspond to parenchymal iron, demyelination, and inflammation. In contrast, using multispectral optoacoustic tomography (MSOT) followed by spectral unmixing has allowed us to extract information about the relative concentrations of oxygenated and deoxygenated hemoglobin for in vivo oxygenation measurements at high spatio-temporal resolutions, and at a relatively lower cost compared to magnetic resonance imaging (MRI) [39]. These characteristics readily make optoacoustic imaging a promising translational technique. Our results suggest that optoacoustic imaging is a promising tool to study the effect of potential treatments on CNS oxygen levels during neuroinflammation.

Previous studies, inspecting the tissue from EAE rats, have shown that breathing normobaric 95% oxygen ameliorates spinal cord hypoxia, decreases demyelination, and improves neurological symptoms [13, 14]. Our data show a similar reversal of hypoxia, acutely in the spinal cord of EAE in vivo already 20 min after breathing 100% oxygen. Additionally, we observed that the increase in oxygen saturation, which went up in both control and EAE mice, did so more slowly in EAE, indicating that the vasculature is somewhat compromised. It also became evident from our MSOT readings that the ventral half of the lumbar spinal cord was most vulnerable; it was most affected by hypoxia and responded to oxygen administration to a lesser extent than the dorsal half. This is interesting because several studies have speculated that hypoperfusion may predispose to hypoxia in the CNS areas with poorest blood supply, given that MS lesions tend to occur predominantly in watershed areas [12, 23, 42, 45]. Indeed, the ventral part of the spinal cord is exclusively supplied by the anterior spinal artery, in comparison with two posterior arteries in the dorsal half, and the irregular augmentation of the anterior spinal artery’s system results in watershed areas that may be vulnerable to hypoperfusion [37].

We also investigated whether alterations in the angioarchitecture of the lumbar spinal cord offer a plausible explanation to the in vivo changes observed in the spinal cord of EAE. Although disturbances of the blood–brain barrier (BBB) are a well-known feature of EAE [18, 26], the open debate on vascular density is still unresolved. Studies carried out using histological techniques and conventional casting show a loss of integrity of the vessels during EAE [4, 7], while others show an increase in vascular density and angiogenesis markers [36, 38]. We have assessed the vasculature of the spinal cord in a volumetric and minimally invasive way by labeling the whole perfused vasculature in vivo with lectin, clearing the spinal column and imaging it with LSFM. In addition, the vessel segmentation and analysis pipeline VesSAP has allowed us to carry out an unbiased and automatic quantification of the angioarchitecture of the spinal cord [40]. Here we report a reduction in the perfused vascular network and network complexity in the lumbar spinal cord during the acute phase of EAE, including a decrease in perfused vessel length, number of bifurcations, and vessel diameter. Finally, we also confirmed by means of immunohistochemistry that our results reflected reduced perfusion and not a loss of vascular density. We revealed that although the blood vessel density is slightly higher in EAE, like previous studies have proposed [8, 20, 24], a significant percentage of the vessels are not perfused. This supports the idea that while spinal cord hypoxia probably activates an angiogenic response to increase vessel density [20, 36, 38], localized inflammation in the spinal cord causes occlusion of part of the vasculature, hindering the perfusion of the vessels, which could be the basis for the decrease in blood flow and hypoxic state that we have described. In line with these results, Mori et al. observed a partial occlusion of the vessels at the lumbar spinal cord during the peak phase of EAE using MRI [29]. In addition, a SWI study has shown venous lumen shrinking in the cerebral vasculature in MS patients [16]. One potential explanation for such shrinking could be that an initial breakdown of the BBB triggers a leak of serum proteins into the CNS parenchyma, causing edema and leading to compressed microvasculature [18, 23]. Another plausible explanation could be that the BBB breakdown causes loss of essential cells on the vessel wall, such as pericytes. Recent discoveries have described a system in which pericytes conform precapillary sphincters that control cerebral blood flow, which could be compromised during neuroinflammation [21].

The strengths of this study include the use of state-of-the-art and minimally invasive imaging techniques and the robust effects found in oxygenation and vascular changes in EAE. Limitations include the use of SJL/J mice, in contrast to the more broadly used C57BL/6 J mice. This is a limitation of the MSOT equipment used, which presents difficulties imaging through more pigmented skin. Furthermore, measurements of the blood flow would have added more information to our study, but such measures in EAE can also be found in the literature [13, 25]. Lastly, measuring the oxygen levels in the grey vs white matter would have been of great interest to identify the functional regions most affected by hypoxia. However, the resolution of our MSOT system was not high enough to distinguish between grey and white matter in a way that we deemed was accurate enough. Nowadays, high-end optoacoustic systems that provide higher resolution imaging are being developed. We welcome further studies that endeavor to confirm and build upon our findings to shed light on the important question of how inflammation affects neurovascular health.

## Conclusions

Our study reports that the inflamed spinal cord is hypoxic during EAE, most likely due to alterations in its vascular network. By using novel imaging modalities, we add a new perspective to a growing body of research interested in vascular and metabolic disturbances in EAE and MS. This study opens up the possibility for future optoacoustic imaging studies to further investigate the connection between neuroinflammation and vascular changes.

## Data Availability

Raw data can be accessed upon reasonable request by contacting the corresponding author.

## Abbreviations

MS: Multiple sclerosis
CNS: Central nervous system
EAE: Experimental autoimmune encephalomyelitis
MSOT: Multispectral optoacoustic tomography
LSFM: Light sheet fluorescence microscopy
OE-OT: Oxygen enhancement during optoacoustic imaging
VesSAP: Vessel segmentation and analysis pipeline
BBB: Blood brain barrier
PLP: Myelin proteolipid protein
DPI: Day post immunization
PBS: Phosphate buffered saline
PFA: Paraformaldehyde
iDISCO+: Immunolabeling-enabled three-dimensional imaging of solvent-cleared organs
SO_2_^MSOT^: MSOT-derived oxygen saturation
HbT: Total hemoglobin
